# Prolonged Hypoxia Increases Survival Even in Zebrafish (*Danio rerio*) Showing Cardiac Arrhythmia

**DOI:** 10.1371/journal.pone.0089099

**Published:** 2014-02-14

**Authors:** Renate Kopp, Ines Bauer, Anil Ramalingam, Margit Egg, Thorsten Schwerte

**Affiliations:** Institute of Zoology and Center of Molecular Biology, University of Innsbruck, Innsbruck, Austria; Scuola Superiore Sant'Anna, Italy

## Abstract

Tolerance towards hypoxia is highly pronounced in zebrafish. In this study even beneficial effects of hypoxia, specifically enhanced survival of zebrafish larvae, could be demonstrated. This effect was actually more pronounced in *breakdance* mutants, which phenotypically show cardiac arrhythmia. *Breakdance* mutants (*bre*) are characterized by chronically reduced cardiac output. Despite an about 50% heart rate reduction, they become adults, but survival rate significantly drops to 40%. Normoxic *bre* animals demonstrate increased hypoxia inducible factor 1 a (Hif-1α) expression, which indicates an activated hypoxic signaling pathway. Consequently, cardiovascular acclimation, like cardiac hypertrophy and increased erythrocyte concentration, occurs. Thus, it was hypothesized, that under hypoxic conditions survival might be even more reduced. When *bre* mutants were exposed to hypoxic conditions, they surprisingly showed higher survival rates than under normoxic conditions and even reached wildtype values. In hypoxic wildtype zebrafish, survival yet exceeded normoxic control values. To specify physiological acclimation, cardiovascular and metabolic parameters were measured before hypoxia started (3 dpf), when the first differences in survival rate occurred (7 dpf) and when survival rate plateaued (15 dpf). Hypoxic animals expectedly demonstrated Hif-1α accumulation and consequently enhanced convective oxygen carrying capacity. Moreover, *bre* animals showed a significantly enhanced heart rate under hypoxic conditions, which reached normoxic wildtype values. This improvement in convective oxygen transport ensured a sufficient oxygen and nutrient supply and was also reflected in the significantly higher mitochondrial activity. The highly optimized energy metabolism observed in hypoxic zebrafish larvae might be decisive for periods of higher energy demand due to organ development, growth and increased activity. However, hypoxia increased survival only during a short period of development and starting hypoxia before or after this phase reduced survival, particularly in *bre* animals. Thus, the physiological plasticity, which enables zebrafish larvae to benefit from a hypoxia, occurs only within a narrow developmental window.

## Introduction

Water living animals are frequently exposed to hypoxic conditions [1] and their ability for complex physiological acclimation, to enable their survival, has been reported in recent years [2]. As oxygen uptake is achieved by bulk diffusion through the skin [3] [4] zebrafish larvae can be raised in an atmosphere even containing 1–5% CO by which hemoglobin oxygen transport is totally blocked [5] [3] [4]. Moreover, resistance of fertilized eggs towards total anoxia has been described [6] [7] though a developmental decrease in the ability to tolerate anoxia was demonstrated by Yaqoob and Schwerte (2010) [8]. Tolerance towards hypoxic conditions is after all known to be highly pronounced in zebrafish, but beneficial effects of hypoxia, increasing even survival in zebrafish have not been reported so far.

Hypoxia inducible factors (Hif isoforms) transcriptionally regulate a high number of genes involved in energy metabolism, angiogenesis and apoptosis and therefore play a central role in the cellular response to hypoxia. Post-translational modifications regulate the degradation rate and transactivation activity of Hif in an oxygen dependent manner (in [9]). The hypoxic signaling of wildtype zebrafish larvae has been characterized only recently [10] [11]. *Bre* larvae, which have a chronically reduced cardiac output, unexpectedly revealed hypoxic signaling under normoxic conditions, as shown by increased levels of hypoxia inducible factor 1 a protein (Hif-1α) concomitant with cardiovascular acclimation [12]. Due to sufficient oxygen bulk diffusion through the skin Hif-1α signaling had to be switched on oxygen independently. Hence it was proposed that the Hif-1α accumulation in *bre* animals might derive from the increased blood cell concentration and consequently enhanced shear stress within the vessels. The latter of which was reported to be closely linked to the endothelial nitric oxide production [13] which in turn is known to inhibit the ubiquitin proteasome.

The *bre* phenotype is caused by the mutated *zebrafish ether-a-go-go-related gene* (*zerg*) coding for a rapidly activating delayed rectifier potassium channel, which is mainly expressed in the ventricle [14]. With this mutated potassium channel ventricular repolarization is significantly prolonged and the heart typically beats in a 2∶1 rhythm so that ventricular contraction is observable only after every second atrial contraction [14] [15]. This phenotype is not continuously expressed, but due to an AV block these mutants show severe bradycardia, even during regular contractions. Therefore, they are characterized by a chronically reduced cardiac output [15] [12]. As convective oxygen transport is not required for developing larvae, *bre* mutants develop into adulthood although mortality during larval development is higher than in control larvae [15]. Hence, it was hypothesized that under hypoxic conditions, when additionally oxygen diffusion through the skin is reduced, proper development of the *bre* zebrafish might be more impaired. Preliminary studies of testing the effect of hypoxia on *bre* survival, contrarily revealed, that *bre* animals were able to cope with hypoxic conditions very well. Consequently, the present study was set out to address physiological acclimation to hypoxia, which might explain the observed beneficial effects of hypoxic treatment on zebrafish larvae.

For proper development and thus survival a balanced energy status is crucial. Hence, oxygen consumption and oxygen supply should be well adjusted. Hypoxia reduces the availability of oxygen, which has to be counteracted by both: enhanced oxygen uptake / distribution mechanisms and reduced oxygen consumption. The main physiological circuits affected by hypoxia are the cardiovascular system and metabolism. We therefore concentrated on cardiovascular performance and convective oxygen carrying capacity on the one hand and analyzed crucial steps of metabolic processes on the other. The mitochondrial density for instance determines oxygen diffusion distances and therefore can enhance oxygen distribution in the cells, cytochrome c oxidase activity correlates with ATP synthesis and represents a sensor for cellular energy levels together with AMP-activated protein kinase (AMPK), which becomes phosphorylated when ATP production should be enhanced. In addition, activity and growth give an indication about the metabolic status of an organism as they contribute to oxygen consumption. By investigating a broad spectrum of relevant molecular and physiological parameters we are able to explain to a large extent the beneficial effect of hypoxia on the survival of zebrafish larvae within a narrow frame of high developmental plasticity.

## Materials and Methods

Animal experiments were performed according to animal ethics permission GZ 66.008/4-BrGT/2004 of the Austrian Federal Ministry for Education, Arts and Culture.

### Laboratory Animals

For all experiments control wildtype (new fish from a local supplier continuously reconditioned control zebrafish stocks) and *bre* zebrafish (*Danio rerio*) were used. Adult homozygous *breakdance* mutants of zebrafish have been obtained from the Max-Planck Institute of Developmental Biology in Tübingen (Artemis Pharmaceuticals GmbH, Cologne, Germany). All parent animals were kept in a fish tank of 50 liters at a temperature of about 26°C.

Larvae were raised in a temperature controlled water bath (28°C) under a 14/10 hours light/dark cycle. Air bubbles and consistent water inflow assured a 100% water oxygenation. For hypoxic exposure (PO_2_ = 5 kPa) PO_2_ was measured by an optic oxygen probe (PreSens GmbH, Regensburg, Germany) and adjusted via a software controlled (LabView, National Instruments, Austin, TX, USA) nitrogen oxide inflow.

### Sampling

As survival is time frame dependent, sampling was performed during periods with highest survival rates. Incubation started at 3 dpf and lasted until sampling. Samples (normoxic control and *bre*, hypoxic control and *bre*) were taken at 3 dpf before starting hypoxia, at 7dpf when survival started to differ and at 15 dpf when survival became more stable. For protein expression analysis additional samples were taken 24 hours after starting hypoxic incubation since then Hif-1α protein reaches maximal expression[10]. All samplings were done at the same daytime (3 hours after lights went on).

### Recording of survival rate

Independent batches of 100 individuals were observed (25 times normoxic controls, 25 times normoxic *bre*, 18 times hypoxic control, 18 times hypoxic *bre*). Every day dead larvae were removed and the fraction of surviving larvae calculated. To exclude acute effects of hypoxia for each group the amount of living 4 dpf old zebrafish larvae was set as 100%.

### Body length measurements

Independent batches of 100 individuals were synchronously fed identical quantities. Whole body length was measured via a binocular microscope containing a length scale. The distance from the most cranial point of the head to the most distal point of the caudal fin determined body length, which was averaged over 12 animals of each group and each sampling.

### Video Imaging of cardiac parameters

#### Preparation

12 larvae of each group were anesthetized with tricaine (0.1 g MS-222 L^−1^ tap water, pH 7.0, Sigma-Aldrich, St. Louis, MO, USA) and embedded in low melting point agarose (including 1.5 mg ml^−1^ MS-222, Sigma-Aldrich, St. Louis, MO, USA). It is known that cardiac performance is not influenced by the anaesthetic or by the oxygen permeable agarose (Schwerte and Fritsche, 2003). The incubation chamber was fixed to a temperature controlled microscope stage set to 28°C.

#### Recording

An inverted microscope (Leitz DM IL, Leica Microsysteme Handelsges. m. b. H., Wetzlar, Germany) was linked to a digital high-speed video camera (HotShot, NAC Image Technology, Simi Valley, CA, USA). As camera configuration tool and for the recording of the single digital picture sequences HSSC link 1.1 software (NAC Image Technology, Simi Valley, CA, USA) was used. Video images from the heart and the caudal part of the *aorta dorsali*s (500 or 300 images, 100 frames per second) were recorded using a 32-fold magnification. Heart rate was evaluated by triplicately measuring time for 30 beats.

#### Analysis

Images were analyzed using the software package Optimas 6.5 (Media Cybernetics, Bethesda, MD, USA). Mean end-diastolic and end-systolic volume of the ventricle was determined as described in detail in previous studies (Hou and Burggren, 1995; Jacobet al., 2002). The ventricular perimeter was outlined at end-diastolic and end-systolic states. Data were analyzed with a “fit-to-ellipse” algorithm calculating the centre of mass and subsequently the best fitting ellipse. The major and minor axis were used to calculate ventricular volume using the formula for an ellipsoid (V = 4/3 _*_ πab^2^). For analysis three diastoles and three systoles per animal were surveyed.

Series of images of the dorsal aorta behind the anus (Schwerte et al., 2003) were taken. In a snap-shot a rectangular area of interest marked between the inner outlines of the vessel. Its volume was calculated assuming a cylindrical shape of the vessel (V = r^2^π _*_ length of the vascular section). All red blood cells within this section were counted and related to its volume. This was done three times per animal.

The convective oxygen carrying capacity was calculated as the product of cardiac output and red blood cell concentration.

### Quantitative Real Time PCR

#### Sampling

For all experimental groups three biological replicates were examined. Time of sampling was three hours after start of day phase.

#### RNA preparation

Total RNA isolation was performed by phenol-chloroform extraction with Trizol Reagent (Invitrogen™, Carlsbad, CA, USA) [12]. Per sample 10 zebrafish larvae were lyzed according to Chomczynski and Sacchi (1987) [16]. Residual DNA was removed with DNAseI (Deoxyribonuclease I, Fermentas International Inc., Burlington, Ontario, Canada) and quality checked by electrophoretic separation (1% agarose gel in TBE buffer). Total RNA concentration was detected fluorometrically using the maximum fluorescence plate reader (VICTOR™ X4, PerkinElmer, Waltham, MA, USA) and Quant-iT™ RiboGreen® (Invitrogen™, Carlsbad, CA, USA) performing measurements in triplicates. cDNA was synthesized according to manufacturer's manual applying Revert Aid™ H Minus M-MuLV reverse Transcriptase (Fermentas International Inc., Burlington, Ontario, Canada). For mRNA measurements SYBR®Green PCR Master Mix (Applied Biosystems, Foster City, CA, USA) was applied. By the means of Primer Express 3.0 software (Applied Biosystems, Foster City, CA, USA) specific primers were designed:

Cytochrome c oxidase 1 (*cox1*) (Entrez Gene ID 140539)

forward 5′-CCCCCCTACCACACATTTGA-3′,

reverse 5′-CCTTCCTTTCTCGTTAATTTGATTG-3′

Uncoupling protein 4 (ucp1) (Entrez Gene ID 83908)

forward 5′- CGTGGTGAAGTACAGCGAGTGA-3′,

reverse 5′- GGGCGCCTTTCTGAACATC-3

pyruvate dehydrogenase kinase isoenzyme 2 (Entrez Gene ID 393971)

forward 5′-CGAATTAGCCAATAAACCAACAAA-3′,

reverse 5′-CACACTTCACCTGCATTTCCA-3

RNA was measured in triplicates. Samples without reverse transcriptase added were analyzed as negative control. Non-specific products such as primer dimer were checked by dissociation curves. Using the sequence detection software SDS 1.3 software package (Applied Biosystems, Foster City, CA, USA) data acquisition and analysis were performed. To standardize differences in the efficiencies of single cDNA syntheses the number of mRNA copies was normalized to 10 ng total RNA. Since a reliable housekeeping gene has not yet been described for early developmental stages of zebrafish [17] and as common housekeeping genes show variations in their mRNA concentrations due to hypoxia [18] [19] no housekeeping gene was used for normalization but RNA quantity and quality precisely checked. Reproducible results approved reliability.

### Microarray analyses

Affymetrix GeneChip analysis was performed and raw expression values normalized and summarized using the CARMAweb analysis program as published in Kopp et al. (2010) [10].

### Cytochrome oxidase DNA content

For mitochondria quantification the amount of cytochrome c oxidase 1 (*cox1*) DNA was measured [20]. For all experimental groups three biological replicates with 10 larvae each were examined. Time of sampling was always three hours after start of day phase. Total DNA isolation was performed by phenol-chloroform extraction using Trizol Reagent (Invitrogen™, Carlsbad, CA, USA). By addition of chloroform and subsequent centrifugation DNA was separated and subsequently precipitated with ethanol. Pelleted DNA was washed (0.1 M sodium citrate solution) and re-suspended in DMPC treated water. Residual RNA was removed with RNaseA (New England Biolabs® Inc., Ipswich, MA, USA) and total DNA concentration was determined fluorometrically using a spectrophotometer (NanoDrop 2000, Thermo Scientific, Wilmington, DE, USA) performing measurements in triplicates. Cytochrome c oxidase 1 DNA was absolutely quantified by qRT-PCR (see above). Specific primers were used: *cox1* (Entrez Gene ID 140539) forward 5′-CCCCCCTACCACACATTTGA-3′, reverse 5′-CCTTCCTTTCTCGTTAATTTGATTG-3′. According to the sample DNA concentration 100 to 500 ng total DNA were added for the measurements.

### Mitochondrial Staining

Normoxic control and *bre* larvae were incubated with 500 nM MitoTracker® Red (Invitrogen™, Carlsbad, CA, USA) for three hours and then imaged by using Zeiss LSM 510 Software (Zeiss, Jena, Germany). Emission was observed with 543 nm excitation and a 560 nm long pass emission filter. Stacks were generated and projected to one single image. Representative images are exemplarily shown.

### Western Blot

For all experimental groups total protein of five biological replicates was examined. Per sample 40 larvae were lyzed in lyses buffer containing glycerol (25%), NaCl (420 mmol/l), MgCl_2_ (1.5 mmol/l), EDTA (0.2 mmol/l), Hepes (20 mmol/l), DTT (125 µmol/l) and a protease inhibitor cocktail (aprotinin, leupeptin, pepstatin, sodium vanadate and phenylmethanesulfonylfluoride). Samples were homogenized on ice using an Ultra-Turrax T25 (IKA Labortechnik, Staufen, Germany). Lysates were precipitated over night with trichloracetic acid diluted in acetone, washed with acetone and resuspended in water. Samples were applied for western blotting using standard procedures as previously described (Schmitz *et al.*, 2000). Protein concentrations were accurately determined by a NanoDrop 1000 Spectrophotometer (Thermo Fisher Scientific Inc., Waltham, Massachusetts, USA). 40 µg protein were applied to each lane. Blotting and detection were performed according to Kopp et al. (2011) [10]. For comparing Hif-1α protein expression samples of all groups were loaded on a single blot. Integrated band density was calculated by using ImageJ 1.47 n software (National Institute of Health, USA). Hif-1α expression of 3 dpf old normoxic control animals was set as 1 and fold induction was calculated. Median values of 5 blots were represented as bar graph.

AMPK blotting was done similarly using 5% BSA in TBS-T for blocking and primary AMP-activated protein kinase alpha (AMPKa) and phosphorylated AMPKa (pAMPKa) antibodies from Cell Signaling Technology, Inc. (Dancers, MA, USA) diluted 1∶1000. Samples were loaded duplicate to one blot to allow direct comparison of the amount of unphosphorylated and phosphorylated AMPK. 15 dpf samples were taken out as at this developmental stage no phosphorylated AMPK can be detected [21]. For all other samples integrated band density was calculated by using ImageJ 1.47 n software (National Institute of Health, USA). Percentage of pAMPK to overall expression (AMPK plus pAMPK) was calculated and the average values of 5 blots represented as bar graph.

### Whole-Mount analysis of lipid content

Tingaud-Sequeira et al. (2011) [22] could already demonstrate that NileRed staining is suitable for semi-quantification of lipid droplets. For all experimental groups 8 biological replicates were examined. Nile Red (Invitrogen™, Carlsbad, CA, USA) was dissolved in acetone (500 µg/ml) and subsequently diluted in water (1∶10,000). Larvae were soaked for 30 min, anesthetized in tricaine (0.1 g l^−1^ tap water, pH 7.0; Sigma-Aldrich, St. Louis, MO, USA) and embedded in low melting point agarose (including 1.5 mg/ml MS-222). Images were taken with the Zeiss LSM 510 Software (Zeiss, Jena, Germany). Nile Red emission ratio enables discrimination of different lipids (Diaz *et al.*, 2008). Red emission was observed with 543 nm excitation and 560 nm long pass emission filters and green emission with 488 nm excitation and 505–550 nm band pass emission filters. Using 10×/0.3 Ph 1 magnification stacks of about 15 µm (10 layers) were generated and all layers projected to one single image. For analysis lipid droplets in the heart, liver, mandibular area and along the intestine were compared for color and dimension. Total area of NEFA staining was analyzed by ImageJ 1.47 n software (National Institute of Health, USA).

3 day control animals were treated with 20 nM Troglitazone (Sigma-Aldrich, St. Louis, MO, USA), which promotes adipocyte differentiation [23] or with 50 µM Resveratrol (Sigma-Aldrich, St. Louis, MO, USA), which scavenges lipid peroxyl radicals [24] for 4 days and then analyzed by NileRed staining.

### Lactate concentration assays

Hot Tris buffer (100 mM Tris, 4 mM EDTA, pH 7.75) was added to each sample containing 10 −20 larvae. After homogenization samples were incubated at 100°C (2 min) and centrifuged at 1,000 g. The supernatant was used for enzymatic determination of L-lactate concentration adding 200 µl lactate buffer (2 M glycine, 800 mM hydrazine sulfate, 10.7 M EDTA, pH 7.9) and 20 µM NAD^+^. To exclude autofluorescence samples were excited 20 times by 355 nm. Thereafter lactate dehydrogenase (0.4 M units) was added to start the reaction. After 30 min NADH fluorescence was measured at 460 nm using a maximum fluorescence plate reader (VICTOR™ X4, PerkinElmer, Waltham, MA, USA). Every sample was measured triplicate and each sampling was repeated 6 times. Lactate values were normalized to the amount of genomic DNA.

### Activity measurements

Fish activity was quantified as described [12] using a custom made activity monitor, which consists of a planar infrared illumination (10×75 mW/sr LED, 875 nm, Siemens Semiconductors, Texas Instruments Inc., Dallas, TX, USA) and a water proof CCD camera with a wide range optics (Security-Center TV7041, Conrad Electronics, Hirschau, Germany). Subsequent frames were arithmetically subtracted and the number of dynamic pixels extracted. Artifacts produced by rapid changes in illumination (during feeding and sampling) were eliminated. Activity of 50 animals was analyzed from 3 to 8 dpf then measurements were stopped to avoid biasing the results by dying fish. Feeding was started at 6 dpf and done twice a day (at 3 and 11 hours after the light went on). For total daily activity calculation the graphs of dynamic pixels were integrated by using MATLAB software (MathWorks, Ismaning, Germany). Values were presented as maximal daily activity with 100% equivalent to the state of all animals move continuously.

### Statistics

The acquired data (cardiovascular parameters, qRT-PCR results, pAMPK percentage, total area of NEFA staining) were statistically analyzed using ANOVA on ranks (Kruskal Wallis test) followed by a post hoc test (software package SigmaPlot 12.0, Systat Software Inc., Chicago, IL, USA). Survival data were analyzed using the cox regression proportional hazard model and for statistical analyses of the NileRed data multivariate exploratory correspondence analysis (Chi-squared test) was applied. Significance was accepted only when P<0.05.

## Results

### Hypoxia beneficially influenced survival

**Figure 1:**
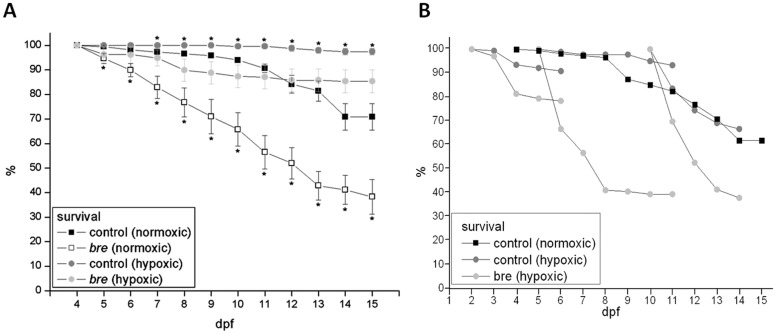
Survival rates. Survival of control and *bre* larvae developing under normoxic or hypoxic conditions from 3 dpf on (A). Survival rate of hypoxic control and *bre* larvae with hypoxic incubation started at different developmental stages (B). Data are shown as mean ± SE. Significance versus normoxic control is marked with *.

Clutches of 100 four dpf old larvae, each of normoxic / hypoxic *bre* animals and normoxic / hypoxic wildtype larvae (Fig. 1A), were screened for the number of dead individuals repeatedly to answer this question.

Already at 5 dpf normoxic *bre* animals showed a significantly lowered survival rate. At 7 dpf a significant increase in survival rate of hypoxic control larvae was observable. At 15 dpf hypoxic control larvae had an about 25% higher survival than normoxic control larvae. Hypoxic *bre* animals never differed from normoxic control larvae. In contrast survival rate of normoxic *bre* animals dropped to about 40%.

The ratio between the individual mortality rates of hypoxic control versus normoxic control animals amounted 11.719, the ratio between hypoxic *br*e versus normoxic *bre* animals 3.176. Mortality rate thus was significantly affected by the oxygen status and genotype.

Starting incubation at other developmental stages (1 dpf, 4 dpf and 9 dpf) never increased survival, which narrowed down a critical time window (Fig. 1B). The later hypoxic incubation was started, the lower survival was.

### Hypoxic exposure of PO_2_ = 5 kPa was appropriate to switch on hypoxic signaling pathways

**Figure 2:**
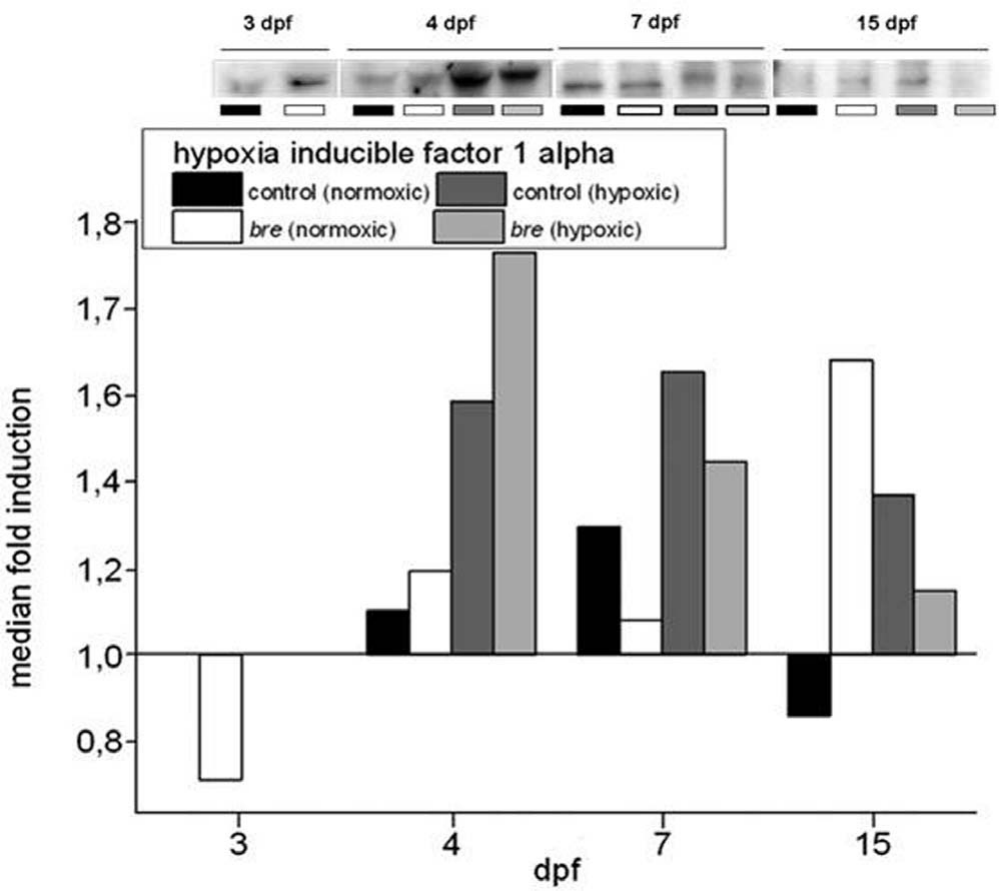
Western blot analysis. HIF-1α protein expression of control and *bre* larvae developing under normoxic or hypoxic conditions. Median fold induction was calculated setting the 3 dpf normoxic control value to 1.

The core signaling protein of the hypoxic signaling pathway is represented by Hif-1α. By western blot analyses its expression in each experimental group over the experimental time was quantified to prove onset of Hif signaling. For comparability the median fold induction was calculated, setting 3 dpf normoxic control values as 1 (Fig. 2).

Normoxic *bre* animals demonstrated an elevated Hif-1α level compared to normoxic control larvae already at 3 dpf. This confirmed previous results [12]. After 24 hours of hypoxia, Hif-1α generally reaches its maximum level [11]. At 4 dpf an increased amount of Hif-1α was seen in all experimental groups. The strongest enhancement was observed in hypoxic *bre* larvae. At 7 dpf also normoxic control fish reached maximum levels. In both hypoxic groups at 7 dpf Hif-1α levels already started to decline, in normoxic *bre* animals Hif-1α levels even dropped below normoxic control values. At 15 dpf normoxic control larvae reached the minimum Hif-1α protein levels, while normoxic *bre* animals showed maximum values. Both hypoxic groups showed an ongoing declining trend.

### Alterations in cardiac activity improved convective oxygen supply of hypoxic zebrafish larvae

To characterize cardiovascular acclimatization for the improvement of oxygen supply heart rate, stroke volume, cardiac output, blood cell concentration, and oxygen carrying capacity were measured and calculated, respectively.

**Figure 3:**
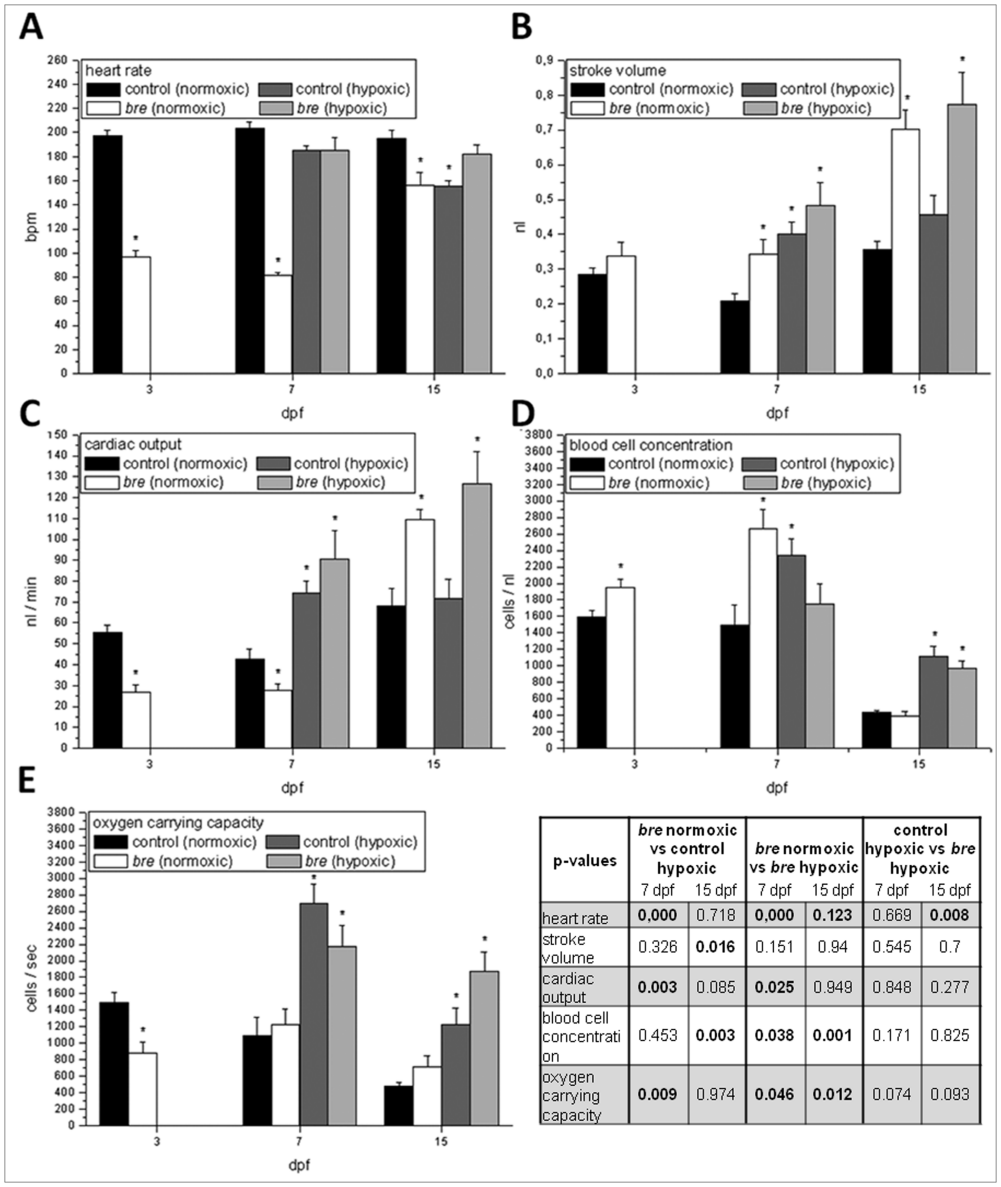
Cardiovascular parameters. Heart rate (A), stroke volume (B), cardiac output (C), blood cell concentration (D) and oxygen carrying capacity (E) of control and *bre* larvae developing under normoxic or hypoxic conditions. Data are shown as mean ± SE. Significance versus normoxic control is marked with *. Significant differences between all other groups are listed in the table.

At 3 dpf normoxic *bre* animals showed a typically reduced heart rate (Fig. 3A). The low values were not compensated by the stroke volume (Fig. 3B). Consequently cardiac output was significantly low (Fig. 3C). The increase in blood cell concentration (Fig. 3D) was not sufficient to keep oxygen carrying capacity high.

At 7 dpf heart rate of normoxic *bre* animals was still significantly reduced (Fig. 3A), while both hypoxic groups did not differ from normoxic control values. Stroke volume was significantly enhanced in both hypoxic groups and normoxic *bre* larvae (Fig. 3B) and the three groups did not differ from each other. Cardiac output was consequently high in both hypoxic groups (Fig. 3C) and low in normoxic *bre* larvae, causing a significant difference between normoxic *bre* animals and all others. Blood cell concentration was increased in normoxic *bre* larvae and hypoxic control animals (Fig. 3D), but no difference was seen between normoxic control and hypoxic *bre* larvae. Hypoxic *bre* animals demonstrated a significant difference compared to their normoxic siblings, but not to hypoxic control larvae. Oxygen carrying capacity was increased in both hypoxic groups (Fig. 3E), the normoxic animals differing significantly from their hypoxic siblings.

At 7 dpf normoxic *bre* as well as both hypoxic groups showed the same increase in stroke volume. In all other measured parameters normoxic *bre* animals differed most, while hypoxic acclimation seemed to be the same in control and *bre* animals.

At 15 dpf heart rate of normoxic *bre* animals had increased, but was still lower than in normoxic control animals (Fig. 3A). The latter was also true for hypoxic control animals. Hypoxic *bre* animals differed significantly from normoxic *bre* as well as from hypoxic control animals. Stroke volume and cardiac output values were high in both *bre* groups (Fig. 3B and C) with an additional significant difference between normoxic *bre* and hypoxic control animals. Blood cell concentration was still high in both hypoxic groups, while normoxic *bre* values had dropped to normoxic control values (Fig. 3D), differing thus from the hypoxic groups. As anticipated, oxygen carrying capacity was elevated in both hypoxic groups (Fig. 3E).

### Effects of hypoxia on cellular energy balance

**Figure 4:**
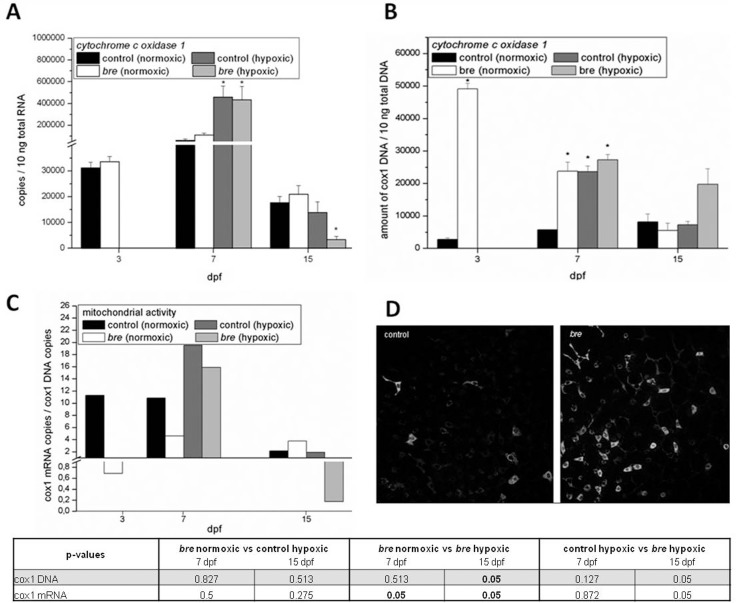
Mitochondrial activity. Cox 1(A) of control and *bre* larvae developing under normoxic or hypoxic conditions. Data are shown as mean ± SE. Significance versus normoxic control is marked with *. Cox1 DNA amount of control and *bre* larvae developing under normoxic or hypoxic conditions (B). Data are shown as mean ± SE. Significance versus normoxic control is marked with *. Significant differences between all other groups are listed in the table. Normalization of cox1 mRNA expression to cox1 DNA content of control and *bre* larvae developing under normoxic or hypoxic conditions (C). Representative images of mitochondrial staining with MitoTracker®Red (D) of normoxic control and *bre* animals (clipping of trunk area).

The transformation of oxygen into usable energy takes place in the mitochondria. Cytochrome c oxidase 1 represents a sensor for cellular energy levels. To gain insight into the mitochondrial activity status, *cox1* mRNA expression was analyzed (Fig. 4A). While both normoxic groups showed similar expression levels, copy numbers of both hypoxic groups exceeded the normoxic values by a factor of up to 6 at 7dpf. At 15 dpf only hypoxic *bre* animals showed different *cox1* mRNA expression as it was significantly reduced.

Since the increased *cox1* mRNA expression on day 7 could be due to an increased number of mitochondria but also to an increase in mitochondrial activity, cox1 DNA content (Fig. 4B), which correlates with the number of mitochondria, was measured. and afterwards normalized to *cox1* mRNA (Fig. 4C). 3 dpf old normoxic *bre* larvae showed a significantly higher amount of *cox1* DNA. At 7 dpf the difference was reduced but still significant. Also both hypoxic groups showed enhanced cox1 DNA values. The increased amount of mitochondria could also be confirmed via MitoTracker® Red staining (Fig. 4D). At 15 dpf only hypoxic *bre* animals reached higher levels than their normoxic siblings. By normalizing *cox1* mRNA expression to cox1 DNA content it appeared that at 3 dpf *bre* animals had less active mitochondria based on a higher number of mitochondria, but a similar *cox1* mRNA expression (Fig. 4D). At 7 dpf activity was still lower than in normoxic control. In contrast both hypoxic groups not only showed a higher number of mitochondria but also higher activity. At 15 dpf the mitochondrial activity level of hypoxic *bre* larvae was obviously low.

**Figure 5:**
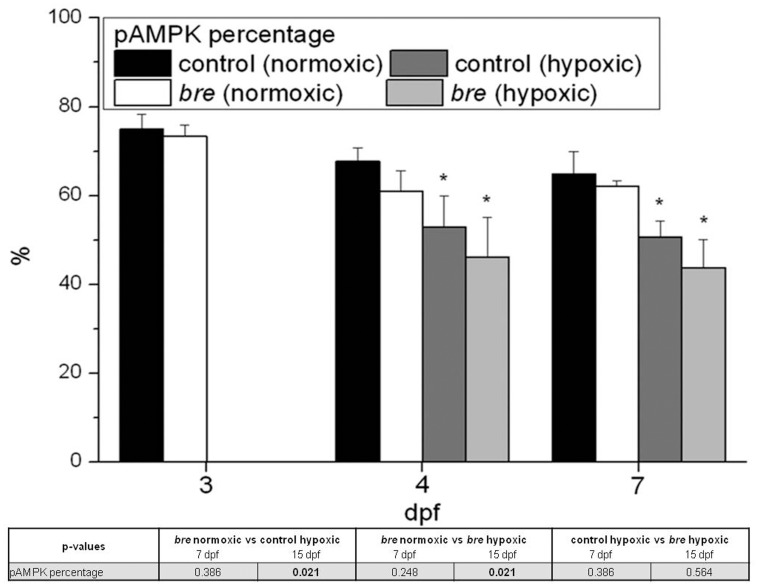
Western blot analysis. AMPK protein expression of control and *bre* larvae developing under normoxic or hypoxic conditions presenting the percentage of pAMPK in the overall AMPK protein expression. Data are shown as mean ± SE. Significance versus normoxic control is marked with *. Significant differences between all other groups are listed in the table.

AMPK plays a crucial role in cellular energy homeostasis. Thus, protein expression of the unphosphorylated and the active phosphorylated form were quantified by western blotting. Calculating the percentage of pAMPK on total AMPK amount gave insight into cellular energy levels. During the whole experimental period normoxic control and *bre* larvae did not differ in their pAMPK / AMPK ratio (Fig. 5). In contrast both hypoxic groups showed a decreased pAMPK percentage.

**Figure 6:**
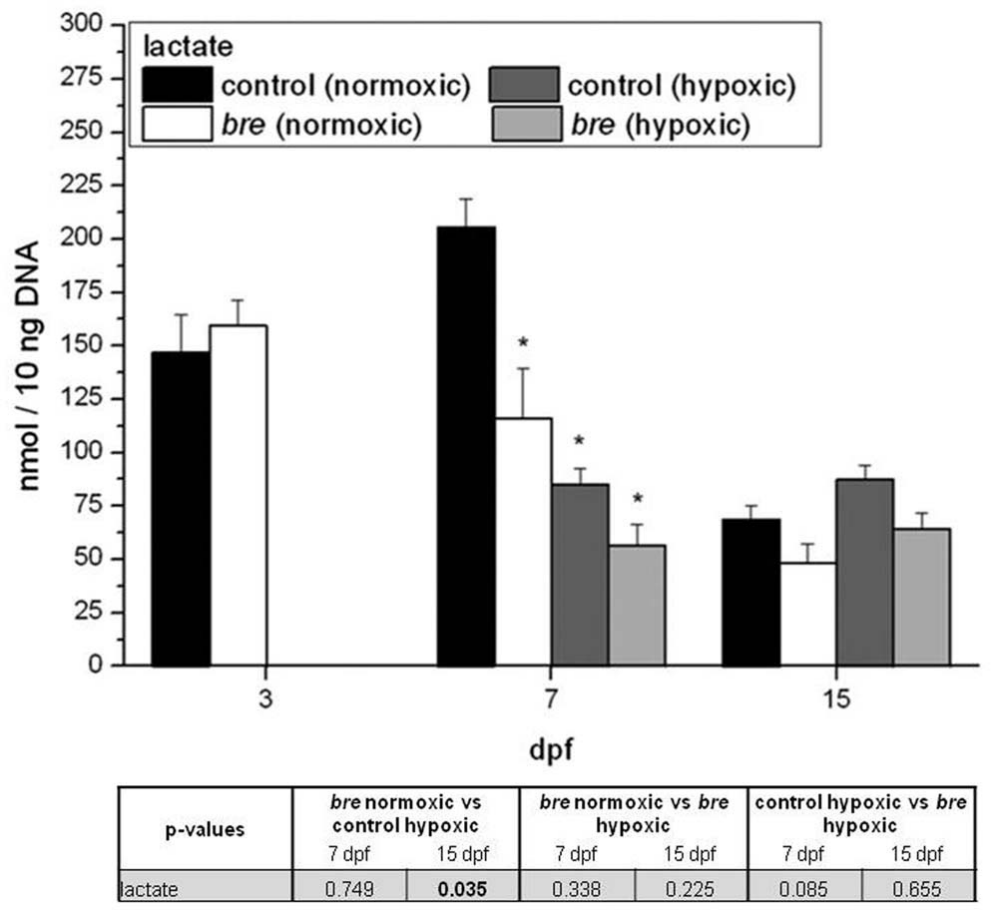
Lactate levels. Lactate accumulation of control and *bre* larvae developing under normoxic or hypoxic conditions. Data are shown as mean ± SE. Significance versus normoxic control is marked with *. Significant differences between all other groups are listed in the table.

Lactate levels are related to the aerobic status of cells as they increase during anaerobic periods. While at 3 dpf and 15 dpf no significant differences were observable in the levels of lactate (Fig. 6),at 7 dpf normoxic *bre* larvae as well as both hypoxic groups demonstrated lower lactate levels than normoxic control animals. At 15 dpf this difference was not shown anymore.

### Energy saving mechanisms were activated during hypoxic exposure

**Figure 7:**
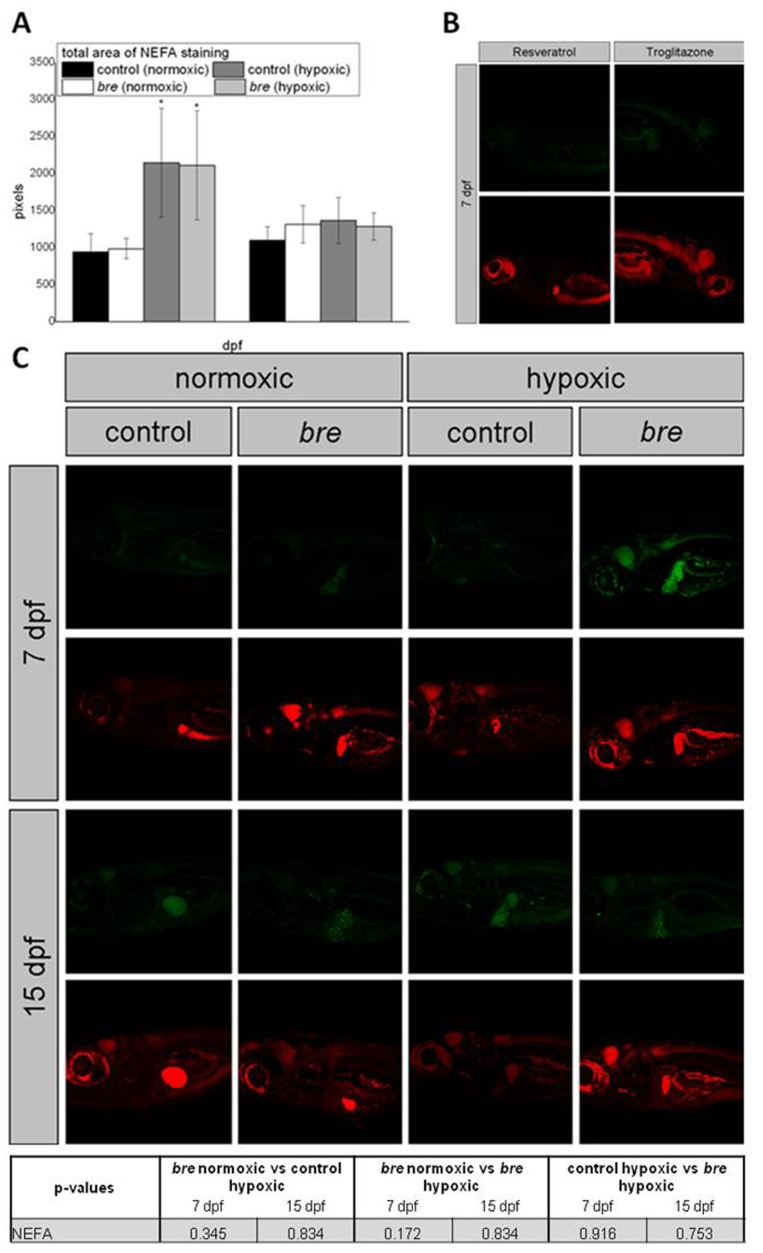
NileRed staining. Lipid accumulation (green - esterified cholesterol, red – NEFAs). Total NEFAs stained are (A) lipid accumulation of Resveratrol or Troglitazone incubated control larvae (B) and of control and *bre* larvae developing under normoxic or hypoxic conditions.

Lipid metabolism represents a metabolic pathway, which consumes most oxygen to produce energy. During a lack of oxygen, a switch to glucose metabolism can be observed. By NileRed staining lipid accumulation was analyzed. The quality of the NileRed staining was proven, as Resveratrol incubated larvae expectedly showed a reduced signaling while Troglitazone incubated animals demonstrated decreased signal intensity (Fig. 7B).

At 7 dpf both hypoxic groups showed an augmented non-esterified fatty acid (NEFA) staining (red) compared to normoxic control larvae. As expected, a chi squared test showed that hypoxia caused significant accumulation of NEFAs along the intestine and the mandibular area (Table 1). Also the total stained area was enlarged (Fig. 7A). In *bre* larvae already under normoxic conditions NEFA accumulation in the mandibular area was detected. Additional staining was seen in the heart and along the intestine. Furthermore, at 7 dpf esterified lipid accumulation (green) along the intestine was observed. In 7 dpf old hypoxic larvae 37% of control and 25% of *bre* larvae showed red signal in the liver while in normoxic control animals only 18% of the animals demonstrated red signaling.

**Table 1:** Statistical analysis of NileRed staining.

chi-square		heart	intestine	liver	mandibular	droplets
**7dpf**	***bre*** ** normoxic**	0.1224	0.4568	0.8666	**0.0002**	0.2563
	**control hypoxic**	0.3451	**0.0412**	0.5066	**0.0000**	0.0607
	***bre*** ** hypoxic**	0.7374	0.3769	0.9058	**0.0000**	**0.0116**

**15 dpf**	***bre*** ** normoxic**	0.3621	0.1260	0.1260	0.6155	**0.0431**
	**control hypoxic**	0.2783	0.6784	0.4319	**0.0452**	0.5336
	***bre*** ** hypoxic**	**0.0249**	**0.0479**	**0.0479**	**0.0024**	**0.0039**

p-values of the chi square test analyzing lipid staining with NileRed. Significance versus normoxic control is marked with bold values. Numbers regarding heart, mandibular area and droplets indicate occurrence of red signaling, intestine and liver indicate preference of red or green signaling.

At 15 dpf normoxic as well as hypoxic *bre* animals showed lipid droplets along the gut. Lipid accumulation in the mandibular region was observed in both hypoxic groups. Most lipid signal was seen in hypoxic *bre* larvae, in heart, liver and intestine. Livers of normoxic *bre* animals showed significant green signaling. 25% of hypoxic control larvae and none of hypoxic *bre* larvae demonstrated a red signal in the liver while in normoxic control animals 41% did.

Activity and growth are highly energy consuming. To see, whether these processes are slowed down during hypoxic exposure, long-term swimming activity was recorded and growth analyzed.

**Figure 8:**
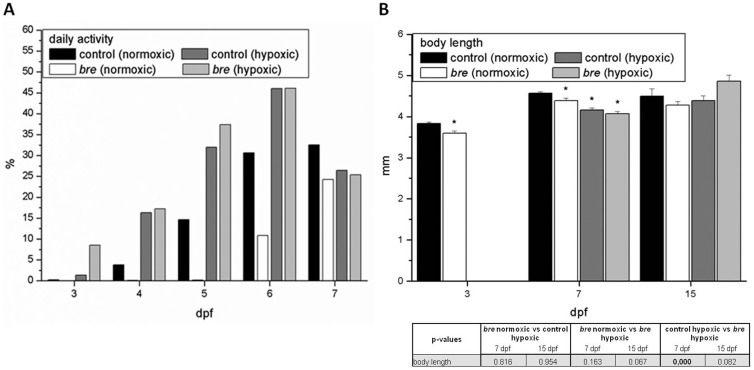
Activity profiles. Total daily activity of 3 to 7*bre* larvae developing under normoxic or hypoxic conditions (A). Body length of control and *bre* larvae developing under normoxic or hypoxic conditions (B). Data are shown as mean ± SE. Significance versus normoxic control is marked with *. Significant differences between all other groups are listed in the table.

Under hypoxic conditions zebrafish larvae demonstrated a classic pattern of day active animals. From 3 to 6 dpf both hypoxic groups showed a higher daily activity (Fig. 8A). Only at 7 dpf activity dropped. Normoxic *bre* animals always represented the most inactive group.

At 3 dpf normoxic *bre* animals were smaller than normoxic control larvae as already shown by Kopp et al. (2010) [12](Fig. 8B). This was also seen at 7 dpf and also both hypoxic groups were smaller. Hypoxic *bre* animals differed significantly from hypoxic control larvae. At 15 dpf all groups were of the same size, with hypoxic *bre* zebrafish showing the highest growth rate (hypoxic control: 5%; hypoxic *bre*: 19%).

### Developmental constrains induced the developmental window, in which hypoxic incubation is beneficial for zebrafish larvae

Affymetrix microarray analyses and therefore general mRNA expression patterns of 3 and 7 dpf old control and *bre* animals should allow the identification of developmental changes, which impede the beneficial effect of hypoxia in older zebrafish larvae.

**Figure 9:**
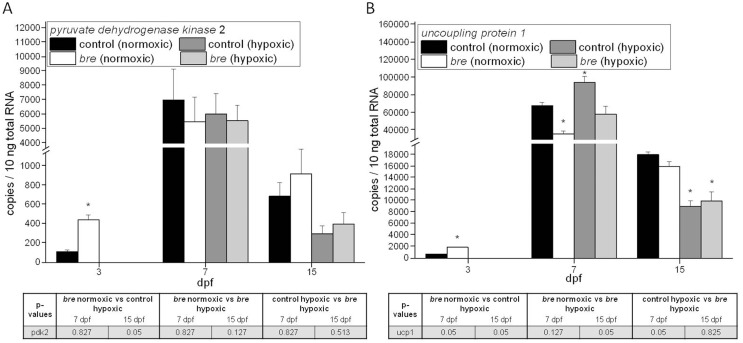
mRNA expression levels. *pdk2* (A) and *ucp1* (B) mRNA expression of control and *bre* larvae developing under normoxic or hypoxic conditions. Data are shown as mean ± SE. Significance versus normoxic control is marked with *.

Comparing gene expression profiles of 3 dpf and 7 dpf old control zebrafish larvae revealed 13 down-regulated transcripts and 80 up-regulated transcripts. In *bre* animals of the same age 517 transcripts were down-regulated and 471 transcripts were up-regulated. 52 transcripts were similarly altered in their expression rate in both groups (Table 2), including *pryruvate dehydrogenase kinase isoenzyme 2* (pdk2), which was up-regulated (M-values for control: 1.61 and for *bre* 4.42) and *uncoupling protein 4* (also referred to as uncoupling protein 1, ucp1), which was also up-regulated (M-values for control: 1.85 and for *bre* 5.12). mRNA expression of both genes was confirmed by qRT-PCR (Fig. 9). The massive modifications in the transcriptome levels observed between 3 and 7 dpf on the one hand and between *br*e mutants and wildtype zebrafish on the other might be the basic to explain the narrow time frame in which hypoxia is beneficial for 3 dpf larvae and even more for *bre* animals.

**Table 2:** Microarray analysis.

Probe	GenBank	UniGene	Description	mean M	
				control	*bre*
Dr.4833.2.S1_at	BI866657	Dr.42614	MAD2 mitotic arrest deficient-like 1 (yeast)	−1,86	4,10
Dr.21634.1.A1_at	AI722397	Dr.21205	zgc:112964	−1,83	5,06
Dr.24379.1.S1_at	AF268044	Dr.24379	cell division cycle 2	−1,72	3,69
Dr.20131.4.S1_at	AW116681	Dr.33492	polo-like kinase 1 (Drosophila)	−1,70	4,00
Dr.3950.1.A1_at	AI884257	Dr.45899	wu:fi25c01	−1,63	3,60
Dr.1691.4.A1_at	BI878593	Dr.28245	karyopherin alpha 2 (RAG cohort 1. importin alpha 1)	−1,57	4,07
Dr.25683.1.S1_at	AL718042	Dr.25683	cathepsin L. b	−1,57	5,46
Dr.10914.1.A1_at	AW567349	Dr.10914	wu:fc49d01	−1,53	2,81
Dr.1557.1.S1_at	NM_173261	Dr.1557	kinesin family member 11	−1,52	3,61
Dr.1050.1.S1_at	BM141327	Dr.40943	zgc:110113	−1,50	3,22
Dr.19153.1.A1_at	AW115530	Dr.31523	wu:fd42f04	1,51	4,83
Dr.26473.1.S1_at	BC053173	Dr.26473	uncoupling protein 2. like	1,51	4,05
Dr.17570.1.S3_at	CD285255	Dr.17570	MAP kinase-interacting serine/threonine kinase 2	1,55	3,42
Dr.23036.1.A1_at	CD014969	Dr.23036	zgc:112282	1,56	4,46
Dr.22139.1.A1_at	AW018965	Dr.22139	wu:fd47e11	1,57	2,21
Dr.4748.1.S1_at	AF273481	Dr.46940	granulin 2	1,60	4,43
Dr.7842.1.A1_at	AW280037	Dr.6698	wu:fj15c09	1,60	3,34
Dr.9528.1.S1_at	BC045993	Dr.9528	pyruvate dehydrogenase kinase. isoenzyme 2	1,61	4,42
Dr.3563.2.S1_at	CD014488	Dr.3563	transmembrane 4 L six family member 4	1,62	3,61
Dr.4937.1.A1_at	AI545488	Dr.4937	wu:fb66a12	1,62	2,71
Dr.11033.1.S1_at	BC046089	Dr.11033	retinol dehydrogenase 1	1,65	2,79
Dr.25253.1.A1_at	CD014618		probable pancreatic proteinase inhibitor	1,66	3,72
Dr.14461.1.A1_at	BI672438	Dr.14461	putative carboxypeptidase S precursor	1,68	4,69
Dr.15507.1.S1_at	AL927888	Dr.15507	cathepsin L.1	1,68	4,84
Dr.2022.1.A1_at	BG891983	Dr.2022	arginase. type II	1,72	4,85
Dr.25219.1.A1_at	CD015412		trypsinogen, cationic precusor	1,74	6,67
Dr.535.2.A1_at	AW116631	Dr.29141	carboxyl ester lipase, like	1,75	7,30
Dr.25222.1.A1_at	CD015551		protein-glutamine gamma-glutamyltransferase K	1,78	5,58
Dr.19704.1.A1_at	AL922331	Dr.34251	zgc:112368	1,79	3,60
Dr.535.1.A1_a_at	AW154740	Dr.535	carboxyl ester lipase, tandem duplicate 1	1,80	7,77
Dr.4905.1.S1_at	BC045464	Dr.4905	uncoupling protein 4	1,85	5,12
Dr.4275.1.S1_at	BM184009	Dr.20720	zgc:66382	2,00	5,83
Dr.4190.2.S1_at	CD015852	Dr.4190	carboxypeptidase A4	2,01	6,90
Dr.24979.1.S1_at	BC051625	Dr.24979	transcription elongation factor B (SIII), polypeptide 3 (110 kDa, elongin A)	2,04	5,22
Dr.18459.1.S1_at	BQ264039	Dr.18459	zgc:92590	2,08	3,32
Dr.4882.1.S1_at	BQ616989	Dr.4882	carboxypeptidase A	2,11	6,12
Dr.4190.1.A1_at	CD015553	Dr.31546	wu:fb66 g10	2,14	6,41
Dr.24991.1.A1_at	CD014897		syncollin	2,17	2,29
Dr.4848.1.S1_at	BC045887	Dr.4848	chitinase, acidic.1	2,17	6,18
Dr.3581.1.S1_at	BM101561	Dr.3581	chymotrypsinogen B1	2,20	6,60
Dr.4959.1.A1_at	AW019487	Dr.31372	zgc:92041	2,27	7,01
Dr.11532.1.S1_at	BC052112	Dr.11532	F-box protein 32	2,27	5,68
Dr.15327.1.A1_at	BM154197	Dr.15327	zgc:92511	2,42	5,58
Dr.18933.1.A1_at	BM316091	Dr.18933	six-cysteine containing astacin protease 3	2,49	7,35
Dr.18599.1.S1_at	BQ479899	Dr.18599	fatty acid binding protein 6, ileal (gastrotropin)	2,54	6,55
Dr.4930.1.A1_at	BI476180	Dr.36424	chymotrypsin-like	2,70	7,38
Dr.5220.1.S1_at	BC044549	Dr.5220	chitinase, acidic.2	2,81	7,20
Dr.4244.1.A1_at	AI544815	Dr.4244	carboxypeptidase A1 (pancreatic)	3,39	7,86
Dr.3581.2.A1_at	CD015432	Dr.26864	chymotrypsinogen 2	3,54	9,50
Dr.24801.1.S1_at	BC042328	Dr.24801	elastase 2	3,81	8,91
Dr.7469.1.S1_at	AY179345	Dr.30551	elastase 2 like	3,86	8,37
Dr.14497.1.S1_at	CD014724	Dr.33926	elastase 3 like	4,23	9,30

Comparison of mRNA expression of 3 and 7 dpf old normoxic control or *bre* larvae. Transcripts, which were up or down-regulated in control as well as *bre* animals, are listed.

## Discussion

### Hypoxia beneficially influenced survival

Zebrafish are known to tolerate hypoxia very well and particularly during early developmental stages this feature is most pronounced. Within the very first hours after fertilization embryos are able to enter a state of developmental arrest during hypoxic conditions. Even after 24 h of anoxia, development can be resumed, if oxygen is available again. Until 4 dpf cardiac re-animation is still possible after 90 min of anoxia-induced suspended animation [8]. At later developmental stages the distinct ability to tolerate very low levels of oxygen is lost [6] [7]. Already in 1998 Burggren established the concept of developmental trajectories and critical windows in developmental physiology. This means that certain time windows allow for the development of particular traits or structures [25]. Following this concept, only during a defined time window sufficient physiological plasticity allows for acclimation to hypoxia, resulting in increased survival rates.

In accordance with these reports hypoxic incubation started at 1 dpf, 4 dpf or 9 dpf in our present study never showed any positive effect on survival. During these periods developmental constrains seemed to reduce plasticity, while at 3 dpf wildtype as well as *bre* mutant zebrafish larvae seemed to be extraordinarily prepared for the hypoxic insult, even profiting from the reduced oxygen availability, as shown by the increased survival rates. To elucidate the underlying mechanisms which enable the animals to such an optimized acclimation to hypoxia at the age of 3dpf a broad spectrum of molecular and physiological parameters of the cardiovascular system and metabolism were investigated and compared to older animals (7dpf) when hypoxic adaptation seemed to be pronounced not that efficiently.

Hif-1α represents the master regulator of the hypoxic signaling pathway. Thus, increased protein expression indicates physiological response to hypoxia. Quantification of Hif-1α protein indicated that the applied persistent hypoxic conditions (5 kPa oxygen) were sufficient to induce Hif-signaling in control as well as *bre* larvae. The observed transient expression was in line with a previous study [10] and implies physiological acclimatization redeeming hypoxic signaling. In hypoxic *bre* animals, Hif-1α expression was even lower than in normoxic *bre* larvae, which show an oxygen independent Hif-1α accumulation[12]. In this context the interference of oxygen independent Hif-1α signaling and environmental hypoxia might cause an increase in natural antisense Hif-1α which was shown to destabilize hif-1α mRNA expression [26] and could therefore account for the reduced Hif protein levels in hypoxic *bre* animals.

A lack in environmental oxygen can be antagonized by enhanced oxygen uptake and transport within the organism. Therefore, hypoxia strongly affects the cardiovascular system. In fish as well as in other vertebrates acclimation to hypoxic conditions is characterized by enhanced cardiac activity, increased red blood cell concentration, improved tissue vascularization, and elevated aerobic metabolic capacity in metabolizing tissues [2].

As expected, in control larvae prolonged hypoxia caused an enhanced cardiac output due to an increased stroke volume, while heart rate was rather reduced. A transient increase in heart rate followed by a continuous reduction appears to be a common response to hypoxic conditions [27]. In this study, it could be shown that apparently prolonged hypoxia regulated stroke volume to enhance convective blood flow. Together with increased red blood cell concentration convective oxygen carrying transport capacity significantly improved.

Previously we were able to demonstrate that even under normoxia *bre* larvae show typical hypoxic acclimation [12]. So, in *bre* mutants stroke volume is remarkably increased, regardless of environmental oxygen conditions. In hypoxic control larvae stroke volume determined the increase in cardiac output, while in hypoxic *bre* animals changes in stroke volume and heart rate enhanced cardiac output. Combined with an increase in red blood cell concentration, oxygen carrying capacity of *bre* larvae was increased by 53% compared to hypoxic control animals, rendering the convective oxygen transport capacity of *bre* mutants highly improved.

Hypoxic incubation significantly increased heart rate in *bre* larvae. These changes can only be explained by altered cardiac channel activities. Even during phases of 1∶1 contraction normoxic *bre* larvae show a mean heart rate of 111.49 beats per minute at 7 dpf[15]. Hypoxic *bre* animals of the same age, however, reached mean heart rate values of 184.91 beats per minute. Recent studies demonstrated that hERG protein expression (*human ether-a-go-go-related gene*) is reversibly down-regulated by prolonged hypoxia [28] [29], which was proposed to occur via the increased generation of ROS in mitochondria [28]. Maturation of hERG protein in turn is inhibited by preventing the binding of hERG to HSP chaperone complexes, which is again mediated by ROS [29]. Moreover, cardiac repolarizing potassium channels, as for example *I*_KATP_ channels which are inhibited by intracellular ATP and therefore provide a link between cellular metabolism and membrane potential [30] [31] open under metabolic stress conditions and shorten action potential durations. Under hypoxia induced metabolic stress these channels therefore might functionally substitute the defective zerg channels.

Therefore, in hypoxic *bre* animals the major improvement was the increase in heart rate, which reached normoxic control level. Together with the mutation related cardiac hypertrophy and increased red blood cell concentration this allowed for coping extremely well with low oxygen levels.

Prolonged hypoxia has been shown to induce characteristic energy saving mechanisms. Metabolism is altered towards a more efficient oxygen use, generating as much ATP without switching to the anaerobic metabolism. The benefit of the improved convective oxygen transport capacity of hypoxic larvae was transferred to the cellular level by an increase in mitochondrial content. Also in normoxic *bre* animals a higher cox1 DNA content was measured, which indicated a higher mitochondrial quantity. Mitochondrial biogenesis has been described as a typical phenomenon induced by hypoxia as mitochondrial hyperplasia reduces oxygen and metabolite diffusion distance inside the cells [32], increases oxidative capacity and reduces average level of oxidative phosphorylation per mitochondria. An increased number of mitochondria can compensate reduced mitochondrial activity and make oxygen consumption more efficient [33]. Additionally, it counteracts possible hypoxia induced cell damage. Low-potential mitochondria [34] can balance respiratory chain activity towards maintaining ATP synthesis and reduce ROS production despite of reduced oxygen availability.

Cox1 is mtDNA encoded and belongs to the cytochrome c oxidase subunit I protein family. This protein family builds up a subunit of the respiratory complex IV, which is the third and final enzyme of the electron transport chain of mitochondrial oxidative phosphorylation. The enzyme activity of Cox1 can therefore denote the energy status of cells. The amount of cox1 DNA indicates the amount of mitochondria. Furthermore, several studies showed that *cox1* mRNA expression correlates with enzyme activity [35] [36]. Thus, normalizing *cox1* mRNA expression to the amount of cox1 DNA gave information about mitochondrial activity (Cox1 enzyme activity per mitochondria). Until 7 dpf normoxic *bre* animals showed less active mitochondria, but a highly increased number of mitochondria. This again indicated the need of maintaining ATP synthesis while reducing ROS production. In hypoxic animals mRNA / DNA ratio was initially aimed towards *cox1* mRNA, but finally *cox1* mRNA expression dropped to control level or and even below, as seen in bre animals.

Electron transfer capacity of hypoxic animals seemed to be highly increased and consequently ATP generation should be augmented. This was also indicated by a lower percentage of phosphorylated AMPK of total AMPK in both hypoxic groups. Oxygen deficiency can stimulate lactate production to at least transiently sustain a certain level of ATP production. For normoxic cells, a basal lactate level has been reported [37]. Particularly, muscle tissue samples contain at least traces of lactate. This might explain the accumulated lactate content in developing normoxic control animals, as muscle tissue proliferates during development. During acute hypoxia, initially stable lactate levels increase [38], [39] [39]. Matching the increased mitochondrial activity, our data even revealed lower lactate contents than in normoxic larvae. So, in chronically hypoxic zebrafish larvae, metabolism might be regulated in a more efficient way, as it has been shown in trained zebrafish larvae, which consume less oxygen while swimming than untrained larvae do [40]. Even humans, chronically exposed to hypoxic conditions at high altitude, manage a given intermediate work load with lower oxygen consumption than lowlanders [41] [42].

Aerobic metabolism can be fuelled by β-oxidation of lipids or glucose. While lipid oxidation is most efficient in terms of ATP production, it requires much more oxygen than glucose oxidation. Accordingly, hypoxia has been shown to cause lipid accumulation in cultured cells [43]. Lipid storage was also significantly increased in 15 dpf old hypoxic *bre* zebrafish, in which esterified lipid droplets along the intestine and in the liver occurred. This could indicate a reduction in lipid oxygenation, probably for the benefit of glucose oxygenation. Increased NEFA concentrations revealed lipolysis due to increased energy demand, albeit hepatic lipid recruitment was higher than oxidation rate. NEFAs sign for lipid mobilization from adipose tissue and represent the major energy source of the liver, where they are oxidized within minutes. NEFA accumulation in various tissues of both hypoxic groups and normoxic *bre* larvae by far exceeded signaling values of control larvae. Moreover, NEFAs signaling in the mandibular region was significantly augmented. Actually, in gills mainly glucose is used as a metabolic fuel [44], but also polar lipid synthesis occurs in teleost gills.

On the bottom line limited oxygen supply should result in less oxygen consumption and therefore reduced activity and growth. In hypoxic control and *bre* animals total daily activity was actually higher than under normoxic conditions, at least until 6 dpf. This was also shown by Egg et al. (2013) [11]. A higher swimming activity can reduce unstirred layers and therefore improve oxygen uptake which seemed to be more relevant than energy saving. Normoxic *bre* animals always showed the lowest activity [12]. On the contrary to control animals, *bre* cardiac output was significantly reduced and therefore blood flow and consequently also nutrition supply were impaired. Thus, probably less energy could be invested in activity.

Growth was initially reduced in hypoxic larvae and normoxic *bre* animals, but at 15 dpf all groups showed the same body length, with a remarkably growth rate in hypoxic *bre* animals and a stagnating on in normoxic control larvae. Also Barrionuevo and Burggren (1999) [45] demonstrated stagnating growth, while simultaneously body mass increased due to enhanced organogenesis. Taken together, energy consumption seemed to be increased in hypoxic zebrafish larvae indicating a higher metabolic efficiency.

### Hypothesis on the benefit of hypoxic acclimation

Developmental effects on metabolism due to growth, increased activity and the switch from yolk utilization to active food intake affect survival rate. Hypoxia in the long run enhanced metabolic efficiency so that zebrafish larvae were prepared for a developmentally increased energy demand, which was reflected by an exponentially increase in total AMPK. Highest survival was seen, when oxygen carrying capacity and metabolic efficiency were high. A crucial difference between hypoxic and normoxic animals was an initially increased mitochondrial activity and low levels of activated AMPK. Although *bre* animals showed Hif-1α expression under normoxic conditions these two parameters were similar to normoxic controls.

The question remains why *bre* larvae can cope so exceptionally well with hypoxic conditions. The major advantage in hypoxic *bre* zebrafish was the extraordinary increase in hear rate. This might be due to hypoxia induced impairment of folding and maturation of the affected cardiac potassium channel [28] [29]. Moreover, cardiac repolarizing potassium channels, like *I*_KATP_ channels, open under hypoxia induced metabolic stress conditions and shorten action potential durations. Therefore, they might functionally substitute the defective zerg channels. The thereby significant increase in heart rate, up to control level, along with the mutation related cardiac hypertrophy and enhanced red blood cell concentration, improved the oxygen carrying capacity enormously. As a consequence transport of nutrients and immune cells should have been improved as well compared to normoxic *bre* animals, which are characterized by a reduced blood flow[12]. The increased heart rate and consequently enhanced cardiac output of hypoxic *bre* larvae, however, cancelled the phenotype and allowed for high mitochondrial activity, crucial for survival.

A critical question arising from this study is: why are the physiological adjustments, triggered by hypoxia, not seen under normoxic conditions, given that they are obviously beneficial for the larvae? Possibly, the production of reactive oxygen species (ROS) prevents these adjustments. In hypoxia adapted larvae, the oxygen transport system is optimized and works more efficiently than under normoxia. Although hypoxia may be frequently encountered in aquatic environments, hypoxia is not persistent. Accordingly, if hypoxia adapted larvae were exposed to longer periods of normoxia, the highly improved oxygen transport system most likely would exceptionally increase internal PO_2_ values, which might results in an inordinate ROS production and consequently cause severe DNA damage [46].

### Hypothesis on the concept of developmental trajectories and critical windows

This concept postulates certain time windows that allow for the development of particular traits or structures [25]. Following this concept, 3 dpf old zebrafish larvae need to have a higher physiological plasticity regarding hypoxic acclimatization than other developmental stages.

To get an idea about what makes 3 dpf old zebrafish larvae so different to other stages, Affymetrix microarrays were analyzed and mRNA expression levels of 3 dpf and 7 dpf old zebrafish larvae compared. Evaluating mRNA expression patterns of control as well as *bre* larvae showed that in both groups 53 transcripts showed a similar expression rate. In context of hypoxia the up-regulation of *pdk2* and *ucp1* seemed to be most interesting.

Up-regulation of *pdk2* mRNA was already shown to be Hif dependent [47]. In turn pdk2 down-regulates the activity of the mitochondrial pyruvate dehydrogenase complex. In 7 dpf old control animals Hif1-α protein expression and therefore *pdk2* mRNA was increased compared to 3 dpf. Thus, a developmentally enhanced energy demand could have caused local hypoxia and therefore Hif signaling, which is also indicated by the drop in haemoglobin oxygen saturation during development [48]. The increasing metabolic stress due to growth and increased activity and the consequently increased oxygen demand might limit physiological plasticity regarding to hypoxic acclimation.

Furthermore, the enormous increase in *ucp1* mRNA expression in 7 dpf old control animals suggests that production of reactive oxygen species (ROS) might increase during early development [49] and present an additional challenge to the animals, rendering them more vulnerable towards an additional environmental lack of oxygen.

As in 3 dpf old zebrafish oxygen delivery reached maximal values (blood cell concentration and oxygen carrying capacity), mitochondrial activity was highest and *pdk2* mRNA expression was lowest, at that developmental stage zebrafish larvae might be better preconditioned regarding to hypoxic acclimation.

However, the increased survival of *bre* mutant animals reported in the present study is also an example of how a deleterious trait might become advantageous under certain circumstances. Thus, a lack of oxygen can alternate the function of the *zerg* mutated potassium channel of *bre* mutants during a certain developmental time frame. It also represents an example, how evolutionary selection forces might generate new features and traits of organisms under particular environmental conditions in specific ecological niches.
